# The SIMM study: Survey of integrative medicine in myeloproliferative neoplasms

**DOI:** 10.1002/cam4.3566

**Published:** 2020-11-03

**Authors:** Krisstina Gowin, Blake T. Langlais, Heidi E. Kosiorek, Amylou Dueck, Denise Millstine, Jennifer Huberty, Ryan Eckert, Ruben A. Mesa

**Affiliations:** ^1^ Department of Hematology University of Arizona Tucson AZ USA; ^2^ Department of Biostatistics Mayo Clinic, Phoenix Phoenix AZ USA; ^3^ Integrative Medicine Program Department of Medicine Mayo Clinic Phoenix AZ USA; ^4^ School of Nutrition and Health Promotion Arizona State University Phoenix AZ USA; ^5^ Mays Cancer Center University of Texas San Antonio TX USA

**Keywords:** essential thrombocytosis, integrative medicine, lifestyle, myelofibrosis, myeloproliferative neoplasms, polycythemia vera, quality of life

## Abstract

Myeloproliferative neoplasms (MPNs) are characterized by significant symptom burden. Integrative medicine (IM) offers unique symptom management strategies. This study describes IM interventions utilized by MPN patients and the association with symptom burden, quality of life, depression, and fatigue adjusted for lifestyle confounders. MPN patients were surveyed online for IM utilization, MPN symptom burden (MPN‐Symptom Assessment Form Total Symptom Score), depression (Patient Health Questionnaire), fatigue (Brief Fatigue Inventory), and a single question on overall quality of life. Measures were compared by IM participation and adjusted for alcohol and tobacco use, BMI, diet, and MPN type using multiple linear and logistic regression. A total of 858 participants were included in the analysis. *A*erobic activity (*p* =< 0.001**)** and strength training (*p* = 0.01**)** were associated with lower mean symptom burden while massage (*p* =< 0.001) and support groups (*p* =< 0.001) were associated with higher levels of symptom burden. Higher quality of life was reported in massage (*p* = 0.04) and support groups (*p* = 0.002) while lower quality of life was noted in aerobic activity (*p* =< 0.001) and strength training (*p* = 0.001). A lower depression screening score was noted in those participating in aerobic activity (*p* = 0.006), yoga (*p* = 0.03), and strength training (*p* = 0.02). Lower fatigue was noted in those participating in aerobic activity (*p* =< 0.001) and strength training (*p* = 0.03) while higher fatigue was noted in those participating in massage (*p* =< 0.001) and breathing techniques (*p* = 0.02). Data available on request from the authors. This international survey of MPN patients on IM usage, has shown that patients who participated in a form of IM had a pattern of decreased levels of symptom burden, fatigue, depression, and higher QoL, as adjusted for health lifestyle practices overall.

## Graphical abstract

This study describes integrative medicine interventions utilized by myeloproliferative neoplasms (MPN) patients and the association with symptom burden, quality of life, depression, and fatigue adjusted for lifestyle confounders. Which, in an internationally diverse MPN patient population and adjusting for healthy lifestyle practices, an overall pattern of lower symptom burden, fatigue, depression, and higher quality of life were revealed with integrative medicine utilization.

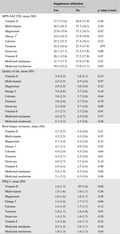

## 1 INTRODUCTION

Myeloproliferative neoplasms (MPNs) including polycythemia vera (PV), essential thrombocytosis (ET), and myelofibrosis (MF) are clonal myeloid malignancies characterized by abnormal hematopoiesis, splenomegaly, risk of thrombotic and/or hemorrhagic complications, acute myelogenous leukemic transformation, and heterogeneous symptom burden.^1^, ^2^ With discovery of the *JAKV617F* mutation in 2005, JAK inhibitors have transformed the treatment landscape for MPN patients, offering new options in therapeutic armamentarium.^3^, ^4^, ^5^, ^6^ Additional novel treatments are under continued development and investigation.^7^ Despite recent pharmacologic advancements, symptoms often persist despite pharmacologic therapy.^8^ In fact, MPN symptoms are frequent and severe, debilitating afflicted patients' quality of life.^9^

Integrative medicine approaches to manage symptoms are becoming increasingly used by patients with cancer and are progressively being accepted and implemented by cancer care providers and organizations.^10^, ^11^ Integrative oncology is defined as a patient‐centered, evidence informed field of cancer care utilizing mind and body practices, natural products and/or lifestyle modifications from different traditions, alongside conventional cancer treatments.^12^ Integrative medicine aims to optimize health, quality of life, and clinical outcomes across the cancer care continuum and to empower patients to prevent cancer and become active participants in their cancer treatment.

Although progress in the field of integrative medicine has occurred, more evidence‐based data to guide clinical practice is needed, particularly in hematologic patients.^13^, ^14^ In this large international survey‐based analysis, we investigated integrative medicine utilization by MPN patients and explore its impact on MPN patient symptom burden.

## 2 METHODS

### 2.1 Study setting

This study was approved by the institutional review board at Mayo Clinic Arizona. The survey titled, “Survey of Integrative Medicine in Myeloproliferative Neoplasms (SIMM)” captured patient demographics, disease‐specific data, comorbidities, and supportive care utilization. Patients were eligible if they were afflicted with PV, ET, or MF. International patients were eligible if they were English proficient. All patients were recruited via social media and email listservs. Informed consent and online self‐report surveys, including measurement of the MPN symptom burden via MPN‐Symptom Assessment Form Total Symptom Score (MPN‐SAF TSS), depression via Patient Health Questionnaire (PHQ)‐2, fatigue via Brief Fatigue Inventory (BFI) Usual, and overall quality of life via a single question assessment were completed via Qualtrics during a 3‐month period in 2016.^15^, ^16^, ^17^

### 2.2 Statistical analysis

Patient surveys with fewer than 10 completed responses were excluded from the analysis. This ensured each patient in the study group had completed at least 10% of the survey. Overall MPN‐specific characteristics are reported. MPN‐SAF TSS, quality of life, PHQ‐2, and BFI were compared between participation in each of the 10 most frequently reported interventions (participation vs. no participation in the intervention). Intervention participation comparisons were adjusted for alcohol consumption (yes/no), smoking status (yes/no), body mass index (BMI) >30 (yes/no), current dietary modifications (yes/no), and MPN disease type (ET, PV, and MF) using multiple linear regression to compare mean values and multiple logistic regression to compare the likelihood of depression. Means and odds ratios (OR) are reported with 95% confidence intervals (95% CI) where appropriate. Other comparisons of frequencies and mean values were conducted using chi‐square tests and *t*‐tests for two‐group comparisons or analysis of variance (ANOVA) for comparisons with more than two groups. *p* values ≤0.05 were considered statistically significant. Analyses were executed using SAS 9.4 (SAS Institute Inc.).

## 3 RESULTS

### 3.1 Patient characteristics

There were 1087 MPN patients consented to complete the survey. Of these, 858 patients completed 10 or more survey responses. There were 338 ET patients, 188 MF patients, 315 PV patients, and 17 other/unspecified. The majority were diagnosed for >3 years (64%) with the remaining participants being diagnosed ≤3 years (35%). The female to male ratio was 3:1. The majority of patients were from the United States (55%), and subsequently the United Kingdom (26%), Australia (3.2%), Germany (3%), and Canada (2.7%). A total of 52 countries were represented. The mean age of the overall group was 58.2 years. Comorbidity prevalence included degenerative joint disease (24%), fibromyalgia (27%), hypothyroidism (15%), restless leg syndrome (11%), chronic fatigue syndrome (9%), obstructive sleep apnea (8%), heart disease (7%), chronic obstructive pulmonary disease (6%), rheumatologic disease (4%) chronic kidney disease (4%), and diabetes mellitus (4%) (see Table 1).

**TABLE 1:** Patient characteristics

MPN patient subtype, *n*	
ET	338
PV	188
MF	315
Other	17
MF DIPSS risk category, %	
Low	8
Intermediate‐1	19
Intermediate‐2	29
High	12
Unknown	32
Time from diagnosis, %	
<3 years	35
>3 years	64
Female to male ratio	3:1
Median age, years	52.8
Geographic location, %	
United States	55
United Kingdom	26
Australia	3
Germany	3
Canada	2
Alcohol use, %	
None	52
1–3 drinks per week	27
4–7 drinks per week	12
>7 drinks per week	9
Cigarette (cigs) use, %	
None	91
<10 cigs per week	4
10–20 cigs per week	2.5
>20 cigs per week	1
BMI, %	
Underweight (BMI < 20)	2
Healthy weight (BMI 20–25)	43
Overweight (BMI 25–30)	33
Obese (BMI >30)	17

Abbreviations: DIPSS, Dynamic International Prognostic Scoring System; MF, myelofibrosis; MPN, myeloproliferative neoplasm.

### 3.2 Integrative modality utilization in MPN patients

Myeloproliferative neoplasm patients utilized a broad spectrum of integrative therapies including aerobic activity (51.5%), massage (28.4%), yoga (25.6%), nutrition (25.2%), strength training (23.8%), acupuncture (19.3%), meditation (19%), breathing exercises (18.4%), chiropractic (16.2%), support groups (14.5%), mindfulness‐based stress reduction (13.6%), walking meditation (12.0%), prayer/religion (10.4%), pet therapy (9.4%), aromatherapy (8.6%), music therapy (8.0%), progressive muscle relaxation (7.0%), guided imagery (6.4%), homeopathy (6.3%), manual therapy (osteopathy/cranial sacral) (6.2%), reiki (5.8%), therapeutic touch (5.7%), Tai Chi (5.2%), art therapy (4.9%), traditional Chinese medicine (4.3%), Qi Gong (3.5%), cooking classes (3.0%), laughter therapy (2.9%), Ayurveda (2.4%), biofeedback (2.2%), dance therapy (2.1%), hypnosis (2.0%), resilience training (0.9%), IV vitamin therapy (0.8%), and narrative medicine (storytelling) (0.3%). Educational resources (32.2%), insurance coverage (35%), referrals from provider (29.4%), and integrative care clinics (27.5%) were deemed useful to continue promote integrative medicine use in conventional care.

#### 3.2.1 Nutrition interventions

Only 24% of patients reported receiving nutrition advice while under care for their MPN. For those that received advice, recommendations were given from a hematologist (10.7%), a registered dietician (7.5%), a primary care physician (6.9%), the intranet (4.2%), friends or family (2.1%), a naturopathic physician (2.0%), a wellness coach (0.8%), television (0.2%), or other sources. Most patients reported satisfaction with the advice they received (85%). At the time of survey administration, 47.7% of patients reported modifying their diet. The reasons patients were modifying their diet included general health (27.3%), to manage weight (24.5%), to manage MPN disease course or symptoms (14.9%), or other reasons. Types of diet modification included Mediterranean (19.0%), paleo/high protein/low carbohydrate (8.9%), vegetarian (8.6%), plant based (5.2%), gluten free (5.2%), low fermentable oligo‐, di‐, mono‐saccharides, and polyols (FODMAP) (1.8%), vegan (1.2%), raw (0.6%), or other diets. Efforts to limit the following food items in the diet were described including limiting added sugar in (30.2%), soda or sugar sweetened beverages (26.1%), processed meat (21.9%), red meat (21.0%), fat (14.7%), carbohydrates (12.9%), dairy (12.1%), animal products (9.0%), soy (4.5%), protein (2.0%), and raw foods (1.3%). Efforts to increase consumption of vegetables (31.9%), fruits (26.8%), whole grains (14.6%), fiber (11.7%), and alkaline foods (1.7%) were reported.

### 3.3 Physical activity participation

Physical activity was commonly utilized in MPN patients including aerobic activity (51.5%), yoga (25.6%), strength training (23.8%), Tai Chi (5.2%), Qi Gong (3.5%), and dance therapy (2.1%). Formal exercise training was received by 10.9% of patients originating from physical therapy or a hospital‐affiliated gym (3.7%), personal training (3.7%), health club (2.9%), online or video (1.9%), friends or family (0.9%), or other. The most desirable form of delivery included in‐person (39.6%), community classes (22.2%), online (13.4%), or other. Patients participated in walking or hiking (48.0%), running or elliptical (6.1%), yoga/Pilates/barre (6.1%), golf (2.2%), or high intensity training (1.4%) during the month prior to survey completion. The majority (50.3%) were being treated for MPN during the period of exercise engagement.

### 3.4 Smoking and alcohol utilization

Patients reported in a typical week drinking no alcohol (52.0%), 1–3 drinks (27.0%), 4–7 drinks (12.1%), and >7 drinks (8.8%). During a typical day, the majority of patients did not smoke cigarettes (91%), however, some consumed <10 (4.1%), 10–20 (2.5%), >20 (1.0%), or other tobacco products (1.3%).

### 3.5 Obesity prevalence

In the overall group, 284 patients (33.1%) were overweight (body mass index 25–30) and 146 patients (17.0%) were obese (body mass index ≥30). Subgroup analysis revealed prevalence of overweight and obese patients within ET group (324 patients) as 36.6% and 19.4%, PV group (300 patients) as 35.0% and 18.3%, and MF group (177 patients) as 37.8% and 15.2%, respectively.

### 3.6 Supplement utilization

Natural products were used in the prior 6 months in 162 (48.4%) ET, 80 (42.8%) MF, and 142 (45.2%) PV patients. Overall, 20.6% of MPN patients reported not disclosing their natural product usage with primary treating physicians. See Table 2 for most common supplements taken by MPN patients and Table 3 for supplement association with symptom burden. Patients reported taking supplements to support general health (35.8%), nutritional deficiencies (13.3%), manage MPN symptoms (11.5%), manage non‐MPN symptoms (9.8%), manage MPN disease directly (5.0%), or other reasons. Those who reported omega‐3 fatty acid supplementation had lower mean MPN‐SAF TSS scores (mean 24.4 with supplementation vs. 27.8 without, *p* = 0.03) and lower BFI (mean 4.1 vs. 4.6, *p* = 0.02). Supplementation with vitamin D, multivitamin, magnesium, and calcium did not correlate with symptom burden, quality of life, depression, or fatigue.

**TABLE 2:** MPN patient supplement utilization

Natural product	ET (*N*, % of total)	MF (*N*, % of total)	PV (*N*, % of total)	Overall (*N*, % of total)
Vitamin D	97 (28.7)	51 (27.1)	90 (28.6)	245 (28.6)
Multivitamin	89 (26.3)	42 (22.3)	52 (16.5)	188 (21.9)
Magnesium	79 (23.4)	32 (17.0)	66 (21.0)	181 (21.1)
Omega 3	76 (22.5)	28 (14.9)	61 (19.4)	170 (19.8)
Calcium	47 (13.9)	24 (12.8)	45 (14.3)	118 (13.8)
Turmeric	43 (12.7)	20 (10.6)	38 (12.1)	104 (12.1)
Green tea	40 (11.8)	21 (11.2)	40 (12.7)	102 (11.9)
Vitamin E	22 (6.5)	9 (4.8)	22 (7.0)	55 (6.4)
Medicinal marijuana	8 (2.4)	4 (2.1)	7 (2.2)	20 (2.3)
Medicinal mushroom	4 (1.2)	2 (1.1)	0 (0.0)	10 (1.2)

Abbreviations: ET, essential thrombocytosis; MF, myelofibrosis; MPM, myeloproliferative neoplasm; PV, polycythemia vera.

**TABLE 3:** MPN patient supplement use and association with symptom burden

	Supplement utilization		*p* value (*t*‐test)
	Yes	No	
MPN‐SAF TSS, mean (SD)			
Vitamin D	27.7 (17.6)	26.9 (17.9)	0.56
Multivitamin	26.5 (16.3)	27.3 (18.2)	0.58
Magnesium	27.0 (15.9)	27.2 (18.3)	0.92
*Omega 3* *	*24.4 (16.5)*	*27.8 (18.0)*	*0.03*
Calcium	25.2 (15.7)	27.4 (18.1)	0.22
Turmeric	24.2 (16.4)	27.5 (17.9)	.079
Green tea	24.1 (17.7)	27.5 (17.8)	0.08
Vitamin E	26.1 (15.8)	27.2 (17.9)	0.67
Medicinal marijuana	31.7 (17.7)	27.0 (17.8)	0.25
Medicinal mushroom	39.5 (23.2)	27.0 (17.7)	0.03
Quality of life, mean (SD)			
Vitamin D	3.9 (2.5)	3.6 (2.7)	0.23
Multivitamin	4.5 (2.5)	4.5 (2.6)	0.97
Magnesium	3.9 (2.5)	3.6 (2.6)	0.22
Omega 3	3.4 (2.6)	3.7 (2.6)	0.18
Calcium	3.6 (2.3)	3.7 (2.6)	0.64
Turmeric	3.8 (2.6)	3.7 (2.6)	0.70
Green tea	3.3 (2.6)	3.7 (2.6)	0.09
Vitamin E	3.7 (2.7)	3.7 (2.6)	0.91
Medicinal marijuana	4.6 (2.7)	4.5 (2.6)	0.57
Medicinal mushroom	5.1 (3.2)	4.5 (2.6)	0.48
Brief fatigue inventory, mean (SD)			
Vitamin D	4.7 (2.5)	4.4 (2.6)	0.21
Multivitamin	4.5 (2.5)	4.5 (2.6)	0.97
Magnesium	4.7 (2.4)	4.4 (2.6)	0.33
*Omega 3*	*4.1 (2.5)*	*4.6 (2.6)*	*0.02*
Calcium	4.4 (2.4)	4.5 (2.6)	0.64
Turmeric	4.4 (2.7)	4.5 (2.6)	0.61
Green tea	4.0 (2.7)	3.7 (2.6)	0.10
Vitamin E	4.4 (2.4)	4.5 (2.6)	0.90
Medicinal marijuana	5.6 (1.9)	4.5 (2.6)	0.06
Medicinal mushroom	5.1 (3.2)	4.5 (2.6)	0.48
PHQ‐2, mean (SD)			
Vitamin D	1.6 (1.7)	05 (1.6)	0.66
Multivitamin	1.5 (1.6)	1.6 (1.7)	0.26
Magnesium	1.6 (1.6)	1.6 (1.7)	0.58
Omega 3	1.4 (1.6)	1.7 (1.7)	0.06
Calcium	1.4 (1.5)	1.7 (1.7)	0.12
Turmeric	1.6 (1.7)	1.6 (1.6)	0.91
Green tea	1.4 (1.5)	1.6 (1.7)	0.20
Vitamin E	1.4 (1.6)	1.6 (1.7)	0.32
Medicinal marijuana	2.2 (1.2)	1.6 (1.7)	0.26
Medicinal mushroom	1.8 (1.3)	1.6 (1.7)	0.64

Abbreviations: MPN‐SAF TSS, Myeloproliferative Neoplasm Symptom Assessment Form Total Symptom Score; PHQ‐2, patient health questionnaire.

* Statistically significant.

Patients received their supplement recommendations from personal research including internet, books, and television (24.5%), friends and family (8.5%), primary care provider (12.8%), hematologist (8.5%), alternative medicine provider (7.1%), other physician or clinician (7.0%), or other sources.

### 3.7 Integrative medicine utilization associations

#### 3.7.1 MPN Symptom Burden (MPN‐SAF TSS)

Patients participating in aerobic activity (mean 33.2 vs. 39.7, *p* < 0.001) and strength training (mean 34.0 vs. 37.7, *p* = 0.013) had lower MPN‐related symptom burden. Patients participating in massage (mean 40.5 vs. 35.3, *p *< 001) and support groups (mean 42.3 vs. 36.0, *p* < 0.001) had higher levels of symptom burden. In the overall group, mean MPN‐SAF TSS scores varied significantly across BMI categories (ANOVA *p* < 0.001), with higher scores seen as BMI increased; healthy weight patients (mean 23), overweight patients (mean 24.0), and obese patients (mean 34.6).

#### 3.7.2 Overall quality of life

Higher quality of life was reported in those receiving massage (mean 5.0 vs. 4.6, *p* = 0.04) and support groups (mean 5.4 vs. 4.6, *p* = 0.002). Lower quality of life was noted in those using aerobic activity (4.2 vs. 5.2, *p* < 0.001) and strength training (mean 4.2 vs. 4.9, *p* = 0.001).

#### 3.7.3 Sleep and psychiatric disturbance

The odds of depression (i.e., PHQ‐2 score ≥3) were 40% less likely for those participating in aerobic activity compared to those not participating (OR: 0.60, 95% CI [0.42, 0.86]; *p* = 0.006). Other interventions associated with decreased odds of depression included yoga (OR: 0.61, 95% CI [0.39, 0.94]; *p* = 0.025) and strength training (OR: 0.58, 95% CI: [0.37, 0.91]; *p* = 0.019). Overall depression was reported in 327 patients (40.7%), anxiety in 295 (36.8%), and stress in 264 (33.6%). The PHQ‐2 revealed clinically significant depression (% ≥3) in 23.4% of patients overall. Of those patients receiving mental health treatment in the last 6 months (*n* = 172 overall, 20.9% of total MPN population), 131 patients (81.9%) received medication, 74 patients (52.9%) received counseling, and 6 patients (5.0%) received group therapy. Overall, difficulty staying asleep was noted in 447 patients (52.1%), difficulty falling asleep in 289 patients (33.7%), insomnia in 268 patients (31.2%), and sleeping too much in 159 patients (18.5%) (see Table 4).

**TABLE 4:** MPM patient sleep and psychiatric disturbance

MPN category	ET (*N* = 338)	PV (*N* = 315)	MF (*N* = 188)	Overall (*N* = 858)
Psychiatric disturbance	(% yes)	(% yes)	(% yes)	(% yes)
Depression	43.7	38.1	40.1	40.7
Anxiety	39.4	33.7	36.6	36.8
Stress	37.9	31.7	27.6	33.6
PHQ‐2 (score ≥3)	23.7	22.0	23.6	23.4
Sleep disturbance				
Staying asleep	53.6	48.9	53.7	52.1
Falling asleep	36.4	32.4	29.3	33.7
Sleeping too much	18.6	18.4	18.6	18.5
Insomnia	28.1	35.6	27.1	31.2
Treatment received in last 6 months (*N* = 172)				
Medication, counseling, or/or group therapy (% yes)	19.4	20.1	25.3	20.9

Abbreviations: ET, essential thrombocytosis; MF, myelofibrosis; MPM, myeloproliferative neoplasm;PHQ‐2, patient health questionnaire; PV, polycythemia vera.

Lower fatigue was noted in those participating in aerobic activity (mean 5.1 vs. 5.9, *p* < 0.001) and strength training (mean 5.2 vs. 5.7, *p* = 0.03). Higher fatigue was noted in those participating in massage (mean 6.1 vs. 5.4, *p* < 0.001) and breathing techniques (mean 6.1 vs. 5.5, *p* = 0.02) (see Table 5).

**TABLE 5:** Adjusted intervention comparisons for symptom burden, QOL, depression, and fatigue

Overall (*N* = 858)	MPN‐SAF TSS, mean yes/no	QoL, mean yes/no	PHQ‐2, odds ratio (95% CI)	BFI, mean yes/no
Aerobic activity (*n* = 442)	33.2/39.7^†^	4.2/5.2^†^	0.60 (0.42, 0.86)^†^	5.1/5.9^†^
Massage (*n* = 244)	40.5/35.3^†^	5.0/4.6*	1.05 (0.72, 1.55)	6.1/5.4^†^
Yoga (*n* = 220)	35.1/37.3	4.5/4.8	0.61 (0.39, 0.94)*	5.5/5.6
Nutrition (*n* = 216)	35.5/37.3	4.6/4.8	1.09 (0.71, 1.67)	5.5/5.6
Strength training (*n* = 204)	34.0/37.7*	4.2/4.9^†^	0.58 (0.37, 0.91)*	5.2/5.7*
Acupuncture (*n* = 166)	38.2/36.6	5.1/4.7	0.74 (0.47, 1.17)	5.9/5.5
Meditation (*n* = 163)	35.4/37.3	4.7/4.8	0.62 (0.38, 1.01)	5.4/5.6
Breathing exercise (*n* = 158)	39.5/36.4	5.1/4.7	1.47 (0.95, 2.28)	6.1/5.5*
Chiropractic (*n* = 139)	36.7/37.0	4.8/4.8	.75 (0.46, 1.21)	5.6/5.6
Support groups (*n* = 124)	42.3/36.0^†^	5.4/4.6^†^	1.45 (0.91, 2.31)	6.2/5.5^†^

Note

Results adjusted for alcohol consumption, smoking status, BMI, current dietary modification, and MPN type. Odds ratios show the likelihood of a PHQ‐2 score ≥3 (yes vs. no). Yes: Those who participated in intervention; No: Those who did not participate in intervention.

Abbreviations: BFI, brief fatigue inventory; CI, confidence interval; MPN‐SAF TSS, Myeloproliferative Neoplasm Symptom Assessment Form Total Symptom Score; PHQ‐2, patient health questionnaire; QoL, quality of life.

* *p* value <0.05.

^†^ *p* value <0.01.

### 3.8 Integrative medicine communication

When patients were asked if they felt their needs were “heard” by their provider regarding the use of integrative therapy, 80.2% of the patients reported no. This unmet need was further emphasized by survey responses: “…I have so many physical conditions that none of my Doctors want to look into it further…I feel like I am march in and back out,” “…I asked what lifestyle changes I could make to ease management of my PV…I was told ‘no lifestyle changes make any difference at all,” “I would like to try these therapies but am never given the option to try.”

## 4 DISCUSSION AND CONCLUSION

The treatment of MPNs has revolutionized in recent decades with the approval *JAK* inhibitor therapy and other targeted therapies, leading to improvements in splenomegaly, symptom burden, and even overall survival.^18^ Despite these advancements, the need for IM interventions to address high symptom burden and poor quality of life remain a large unmet need in the MPN patient population. In this study, we sought to better understand current integrative medicine utilization patterns, lifestyle behaviors, and explore associations with MPN symptom burden, quality of life, depression, and fatigue to help direct research regarding future integrative medicine interventions.

In this study, MPN patients utilized a diverse milieu of integrative medicine therapies. The most commonly used therapies included aerobic activity (51.5%), massage (28.4%), yoga (25.6%), nutrition (25.2%), and strength training (23.8%). Supplement use was common with 44.7% of the overall MPN population using supplements in prior 6 months, but this was undisclosed to their treating physicians in 20.6% of cases. Additionally, 80.2% of patients felt their integrative health needs were not heard by their health‐care provider. This underscores the importance of enhancing integrative therapy communication in our health system.

Although nutrition and exercise were commonly used, health‐care directed counseling was infrequent with only 24% of patients receiving nutrition counseling and 10% receiving formal exercise counseling. Despite the reported interest and engagement in aerobic activity and nutrition interventions, a high prevalence of obesity was identified. This highlights the important need of lifestyle counseling within the MPN patient population. Lifestyle intervention may be particularly important for patients afflicted with ET and PV given a patient's cardiovascular risk is contributory to the overall thrombotic risk assessment and cytoreductive therapy decision algorithms.^19^ Additionally, higher MPN‐SAF TSS mean scores were noted in obese and overweight patients in comparison to healthy MPN patients (mean MPN SAF‐TSS 34.6, 24.0, 20.0, respectively), suggesting obtaining healthy body weight may help modulate symptom burden (ANOVA *p* < 0.001). Although, future studies examining lifestyle interventions is needed, this study suggests general lifestyle counseling would be appropriate and likely well received within the MPN community.

In this study, we explored the association of symptom burden (MPN‐SAF TSS score) and QOL (single question assessment) with the 10 most frequently used integrative interventions. Intervention participation comparisons were adjusted for alcohol consumption, smoking status, body mass index, current dietary modification, and MPN disease type to limit confounding factors. Our analysis revealed aerobic activity (*p* < 0.001) and strength training (*p* = 0.013) were associated with lower MPN symptom burden (MPN‐SAF mean score 33.3 vs. 39.7 and 34.0 vs. 37.7, respectively). It is not known if this association is due to patients with lower symptom burden simply being more able to participate in aerobic and strength training, or if participation itself led to lower symptom burden, or if those with high symptom burden just were not able to exercise. Studies evaluating the impact of physical activity in MPN patients are limited and physical activity interventions may be an area of future research in the non‐pharmacological management of MPN associated symptom burden.^20^

In our study, aerobic activity (*p* < 0.001) and strength training (*p* = 0.001) were paradoxically associated with a lower quality of life despite the lower symptom burden observed in this population. This is in contrast to previously published data supporting patient MPN symptom severity as being a good predictor of quality of life.^21^ This association may simply reflect that those with lower quality of life are more likely to seek out exercise‐based wellness strategies. More studies are needed to better understand the effect of exercise interventions on quality of life.

Massage (*p* < 0.001) and support groups (*p* < 0.001) were associated with higher symptom burden, perhaps representing a cohort of highly symptomatic patients seeking out non‐pharmacologic symptom management and social support. Interestingly, higher quality of life was noted with massage (*p* = 0.04) and support groups (*p* = 0.002) despite the high symptom burden (MPN SAF‐TSS mean score 40.5 vs. 35.3 and 42.3 vs. 36.0, respectively). To our knowledge, no prior studies have investigated the effect of massage or support group interventions in MPN patients. In the larger hematologic cancer community, psychosocial support is associated with maintenance of well‐being.^22^ Future interventions utilizing massage and supports groups may be warranted in the MPN patient population, and may be well suited in patients with higher symptom burden.

The association of the most frequently used integrative therapy and depression was also explored in this analysis. The odds of depression (i.e., PHQ‐2 score ≥3) were 40% less likely for those participating in aerobic activity, strength training, and yoga compared to those not participating. There is little prior data on the impact of interventions, pharmacologic, and non‐pharmacologic, on psychosocial well‐being in the MPN patient population.^23^, ^24^, ^25^ Clinical trials are needed for the management of psychosocial dysfunction in MPN patients. Our analysis suggests future trials targeting depression in MPN patients may include interventions including aerobic activity, strength training, and yoga in a multidisciplinary treatment approach.

Fatigue, a frequent and debilitating MPN manifestation, was lower in those participating in aerobic activity (*p* < 0.001) and strength training (*p* = 0.03), or conversely this could be interpreted as those with less fatigue participated more in physical activity. The association of fatigue and physical activity was previously established in an internet‐based survey of MPN patients (*N* = 1676), with moderate/severe fatigue being present more frequently in those patients who did not exercise compared with those who reported exercising at least once per week (*p* < 0.001).^26^ Studies evaluating the impact of exercise on MPN associated fatigue are needed.

Supplements were used in 44.7% of the overall MPN, however; of the top 10 most commonly used supplements, only omega‐3 fatty acid supplementation was found to be statistically significant for impacts on MPN‐SAF TSS (mean 24.4 with supplementation vs. 27.8 without, *p* = 0.03) and lower BFI (mean 4.1 vs. 4.6, *p* = 0.02). No supplement use was found to correlate with improved quality of life or lower depression. The improvement in MPN patient symptom burden and fatigue with omega‐3 fatty acid supplementation is notable and further study of this is warranted. It is unknown what type of omega‐3 fatty acid was supplemented, at what dose, and for what duration it was ingested. These are critical questions that further study should investigate.

While the majority of MPN patients were participating in some form of integrative medicine intervention, when asked if they felt their needs were “heard” by their provider regarding the use of integrative therapy, 80.2% of the patients reported no. This highlights the need for more integrative medicine practitioners and more robust integration of these practitioners and modalities within standard medical care.

Limitations of this study include the nature of survey‐based patient‐reported outcome assessment and a female gender bias. With self‐report survey, the amount of overlap in a given patients disease comorbidities and integrative methods is unknown which may confound the results. Additionally, our analysis evaluated the association of symptom burden with only the 10 most frequently utilized integrative modalities. Less frequently used integrative therapies may offer benefit and should be evaluated in future analysis. While the incidence of overweight and obesity was high in the population, the association of this with type of integrative medicine use was not analyzed. This may be of interest for future study in an effort to design interventional studies for the overweight and obese MPN population. Finally, while correlations are suggestive, they do not prove causality and may be presumptive. Adequately powered randomized‐controlled trials are needed to understand the impact of integrative medicine on MPN symptom burden. Despite these limitations, this study represents the largest, most internationally diverse, and comprehensive evaluation of integrative therapy utilization in MPN patients to date.

In conclusion, pharmacologic therapy has offered major contribution to those afflicted with MPNs. However, the symptom burden of treated patients remains unacceptably high with significant impacts on activities of daily living and quality of life. Integrative medicine may help to alleviate symptoms and enhance quality of life when complementing standard therapies. Future intervention trials employing exercise, diet, massage, support groups, supplements, and other therapies will be integral to our growing understanding of the potential contributions provided by integrative medicine to MPN patients. Additionally, health‐care delivery platforms that enhance integrative therapy communication and counseling should be explored.

## CONFLICT OF INTEREST

Gowin: Incyte Speaker Bureau, Scientific Advisory Board. Mesa: Consultant: Novartis. Research Incyte, Celgene, CTI, Genentech.

## Data Availability

Data available on request from the authors.
